# Update in the Management of ANCA-Associated Vasculitis: Recent Developments and Future Perspectives

**DOI:** 10.1155/2021/5534851

**Published:** 2021-04-08

**Authors:** Karla N. Samman, Carolyn Ross, Christian Pagnoux, Jean-Paul Makhzoum

**Affiliations:** ^1^Vasculitis Clinic, Canadian Network for Research on Vasculitides (CanVasc), Division of Internal Medicine, Hôpital du Sacré-Coeur de Montréal, University of Montreal, Montreal, Quebec, Canada; ^2^Vasculitis Clinic, Canadian Network for Research on Vasculitides (CanVasc), Division of Rheumatology, Mount Sinai Hospital, University of Toronto, Toronto, Ontario, Canada

## Abstract

Significant progress has been made in the treatment of ANCA-associated vasculitides (AAV), notably in granulomatosis with polyangiitis and microscopic polyangiitis. Over the past few years, many innovative studies have changed the way we now induce and maintain remission in AAV; achieving remission while limiting treatment toxicity is the key. This article provides an in-depth, up-to-date summary of recent trials and suggests treatment algorithms for induction and maintenance of remission based on the latest guidelines. Future possible therapies in AAV will also be discussed.

## 1. Introduction

Vasculitides are classified according to the size of vessels predominantly involved in the inflammatory process [1]. Antineutrophil cytoplasmic antibody- (ANCA-) associated vasculitides (AAV) are predominantly necrotizing, small vessel vasculitides and include granulomatosis with polyangiitis (GPA, previously known as Wegener's granulomatosis), microscopic polyangiitis (MPA), and eosinophilic granulomatosis with polyangiitis (EGPA, formerly Churg-Strauss syndrome) [1, 2]. Serological markers include myeloperoxidase- (MPO-) ANCA and proteinase 3- (PR3-) ANCA, previously detected by immunofluorescence as perinuclear (p-ANCA) and cytoplasmic (c-ANCA), respectively [3–5].

Therapeutic advances in AAV have been substantial in the past 20 years. Before the use of high-dose glucocorticoids (GC) and cyclophosphamide (CYC), the mortality rate of patients with severe AAV was up to 80% one year after diagnosis [3]. Recent mortality rates decreased, and the estimated 5-year survival is 74-91% and 45-76% for GPA and MPA, respectively [6]. Adequate medical management is crucial as patients with AAV remain at high risk of relapsing disease [4, 7]. Infections associated with immunosuppressive therapy have become the leading cause of deaths during the first year of diagnosis of severe AAV, contributing to 34-48% of the mortality reported in some studies [3, 8].

This review presents the main clinical trials and advances in GPA and MPA that shaped today's induction and maintenance of remission therapeutic algorithms. This article provides an up-to-date summary of recent trials and suggests treatment algorithms for induction and maintenance of remission. The treatment of EGPA will not be discussed in this review. The present article does not replace guidelines issued by international and national vasculitis societies but is aimed at providing a pragmatic approach to the management of GPA and MPA.

## 2. Classification of Disease Severity

In practice, AAV are clinically classified according to the severity (nonsevere or severe) and the extent (limited or systemic) of the disease [9]. The definition of disease severity varies slightly according to the studies or guidelines; the latter usually define severe AAV as requiring CYC or rituximab (RTX) for the induction of remission. Organ-threatening manifestations usually include severe alveolar hemorrhage, rapidly progressive glomerulonephritis (GN), and severe gastrointestinal, cardiac, central nervous system, or ocular involvement. In clinical trials, the Birmingham Vasculitis Activity score (BVAS) [10] is often used to determine disease activity for GPA and MPA and identify patient with “major” items, corresponding to a severe disease. Furthermore, stratification of the risk of relapse is important in individualizing therapy. Patients with GPA, with a previous history of relapsing disease, and PR3-ANCA are more likely to relapse than patients with MPA or with MPO-ANCA. Other factors may possibly be linked with relapses, such as ANCA status during follow-up, genetic background, levels of T cell activation, and other possible exogenous factors [7].

## 3. Treatment Principles

**Figure 1:**
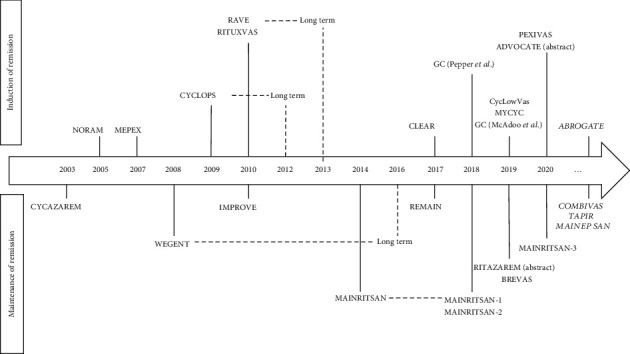
Timeline of hallmark trials of ANCA-associated vasculitis.

Treatment of severe GPA and MPA includes induction of remission, followed by maintenance of remission to prevent disease relapse. The cornerstone induction and maintenance trials presented in this review are summarized in Tables 1 and 2, respectively. These studies are also shown on a timeline according to their publication year (Figure 1) [3, 4, 11–13]. Knowing the older age of the population affected (peak incidence between 50 and 74 years) and the potential drug toxicity, the past years have been marked with multiple clinical trials aimed at finding the most efficient yet safest regimen for GPA and MPA [3, 4].

## 4. Induction of Remission

The remission induction therapy is initiated upon initial diagnosis or relapse of GPA and MPA [4, 11]. The duration of induction of remission is usually between 3 and 6 months, with the objective of preventing mortality, achieving clinical remission, and limiting permanent organ damage [4]. In addition to age and comorbidities, therapeutic regimen choice is guided by disease severity [11].

### 4.1. Induction Therapy of Severe GPA and MPA

**Figure 2:**
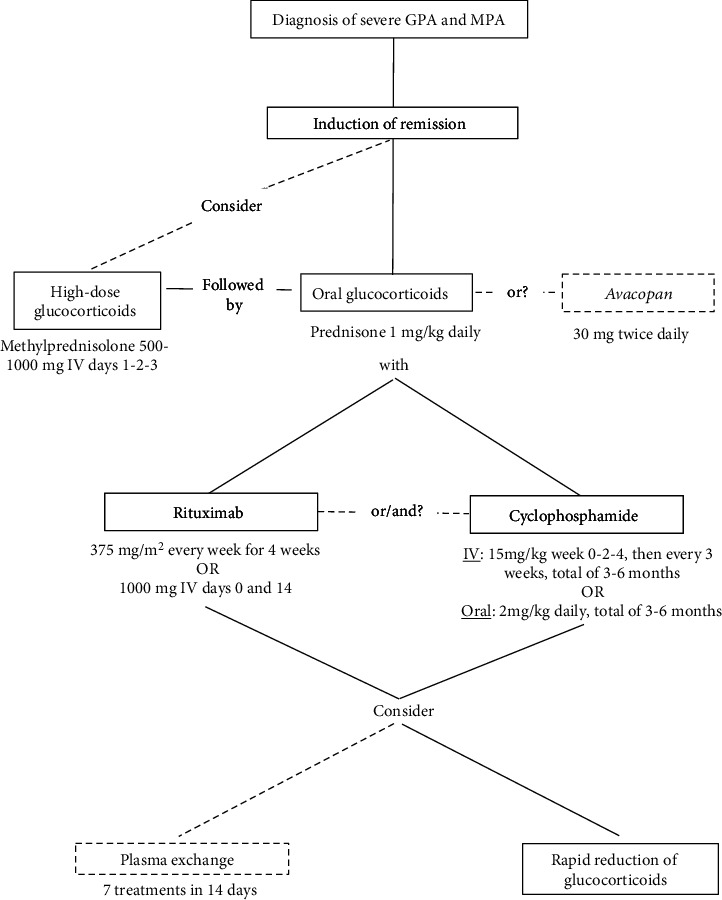
Suggested algorithm of induction therapy in severe GPA or MPA.

The current induction therapy of severe GPA and MPA (Figure 2) consists of GC combined with either RTX or CYC [5, 14–16].

#### 4.1.1. Glucocorticoids

GC are administered in the induction of remission of severe GPA and MPA [12, 14–16]. Intravenous (IV) methylprednisolone pulses (500–1000 mg per day for 1 to 3 consecutive days) are often used for organ- or life-threatening GPA and MPA, followed by oral prednisone at a dose of 1 mg/kg daily [12]. The widely accepted use of IV GC pulses has been questioned. In a retrospective multicenter cohort study [17], patients receiving IV methylprednisolone had a significantly higher rate of infection with no benefit in survival, renal recovery, or relapses. Hence, pulses of IV methylprednisolone may be considered in severe, organ or life-threatening GPA or MPA. However, “evidence is lacking to prove its efficacy and shows potential risk of adverse effects, mostly related to increased incidence of infections and diabetes,” as emphasized in the recently published CanVasc recommendations [5].

The numerous adverse effects of GC have led to many attempts to reduce their doses and accelerate tapering in clinical trials [18]. The Plasma Exchange and Glucocorticoids in Severe ANCA-Associated Vasculitis (PEXIVAS) trial showed that a rapid taper of GC was noninferior to the standard, slower tapering regimen in terms of death or end-stage renal disease (ESRD). At 6 months, the cumulative dose of oral GC in the reduced-dose group was less than 60% of that in the standard group and was associated with less severe infections during the first year of treatment [19]. The combination of CYC and RTX has also been attempted to accelerate GC tapering [20–22], with some studies limiting the duration of GC treatment to 2 weeks, although strong evidence with randomized controlled trials (RCTs) is lacking.

GC tapering may begin 2 to 4 weeks after initiation of immunosuppression for the induction of remission (with CYC or RTX). It is reasonable to aim for a daily oral dose of prednisone (or equivalent) of 7.5 to 12.5 mg at 3 months of remission induction therapy [19].

#### 4.1.2. Cyclophosphamide

CYC has been widely used in combination with GC to induce remission of GPA and MPA; its efficacy has been proven for over 30 years [23, 24]. In combination with high-dose GC, CYC induces remission in up to 75% and 90% of patients, at 3 and 6 months, respectively [7].

CYC can be administered orally or intravenously. The CYCLOPS trial compared daily oral (DO) versus IV pulse CYC in patients with newly diagnosed severe renal GPA or MPA (Table 1). Renal involvement was defined as serum creatinine levels between 150 and 500 *μ*mol/L, proteinuria (over 1 g daily), hematuria, or proven necrotic pauci-immune GN on kidney biopsy. At 9-month follow-up, no significant difference was seen in the time to remission between groups (*p* = 0.59), with a lower cumulative dose of CYC in the IV pulse group (8.2 g versus 15.9 g with the DO CYC; *p* < 0.001) and a lower rate of leukopenia (hazard ratio (HR) 0.41, (CI 0.23 to 0.71)) [25]. However, long-term follow-up of these patients at a median of 4.3 years showed that relapses were significantly lower in DO CYC than IV pulse CYC (20.8% versus 39.5%, respectively; HR 0.50, 95% CI 0.26 to 0.93; *p* = 0.029), but without any difference in mortality (*p* = 0.92) or renal function (*p* = 0.82) [26].

Therefore, CYC may be given intravenously at a dose of 15 mg/kg for each infusion (maximum 1200 mg per pulse) at weeks 0, 2, and 4 and then every 3 weeks for a total of 3 to 6 months. If given orally, the daily target dose is 2 mg/kg (maximum of 200 mg/day) [7, 12]. CYC dosing must be adjusted according to age and glomerular filtration rate (GFR) and subsequently corrected according to the presence and severity of cytopenia.

#### 4.1.3. Rituximab

RTX is a chimeric anti-CD20 monoclonal antibody that depletes B cells. Because of the potential toxicity associated with CYC (cytopenias, infertility, and risk of hemorrhagic cystitis and bladder cancer), RTX has gained popularity for the induction of remission in AAV. Nevertheless, the long-term toxicity of CYC can be minimized by limiting its total lifetime cumulative dose to less 25 g [5, 27].

RTX may be used as a first-line therapy for induction of remission with GC in severe GPA and MPA, especially in patients with contraindications to CYC [5]. Two main RCTs compared CYC and RTX head to head.

The RITUXVAS trial was conducted as an open-label randomized study and included patients with newly diagnosed generalized GPA or MPA with renal involvement (median GFR below 18 mL/min/1.73m^2^). Participants in the CYC group received the drug intravenously for 3 to 6 months, followed by azathioprine (AZA). Patients in the RTX group received 375 mg/m^2^ every week for 4 weeks, with 2 pulses of IV CYC (15 mg/kg) with the first and the third RTX infusion. Comparing CYC to RTX (Table 1), there was no significant difference in sustained remissions (*p* = 0.68), severe adverse reactions (*p* = 0.77), or deaths (*p* = 1.00) at 12 months of follow-up. In RITUXVAS, RTX was equivalent to CYC, but not superior for induction of remission [28].

The Rituximab in ANCA-Associated Vasculitis (RAVE) trial, a noninferiority double-blinded RCT, studied patients with severe newly diagnosed or relapsing GPA or MPA, with positive ANCA and a BVAS/WG > 3. At 6-month follow-up, remission, defined as a BVAS = 0 and a successful complete tapering of prednisone, was noninferior with RTX compared to CYC, respectively, 64% versus 53% (*p* < 0.001 for noninferiority). In patients with relapsing GPA or MPA, RTX was shown superior to CYC (67% versus 42%; *p* = 0.01) for remission induction at 6 months [29]. A longer follow-up of the relapsing patients confirmed that RTX was superior to CYC at 12 months' follow-up (48% versus 39%, *p* = 0.009), but no longer at 18 months (*p* = 0.06). For newly diagnosed and relapsing patients combined, remission at 18 months with RTX was noninferior to CYC (39% and 33%, respectively, *p* < 0.001). However, it is important to note that patients in the CYC received AZA after 3 to 6 months of treatment with CYC, whereas the RTX group received no further treatment [30]. Interestingly, patients with PR3-ANCA-positive antibodies doubled their chances of remission at 6 months with RTX when compared to a matched population receiving CYC [31].

Although the 4-dose RTX regimen (375 mg/m^2^ weekly for 4 weeks) has been most extensively studied for induction therapy in AAV, many clinicians choose to use the 2-dose regimen (1000 mg IV on days 0 and 14, as used for rheumatoid arthritis) to reduce infusion frequency, total dose, and cost. A recent meta-analysis compared the efficacy and safety of these two RTX regimens for the induction of remission in severe AAV [32]. No difference was found in terms of efficacy and safety between the 4-dose and 2-dose RTX regimens. However, head-to-head clinical trials comparing these two regimens have not yet been conducted.

#### 4.1.4. Combination Rituximab and Cyclophosphamide

The CycLowVas induction of remission cohort study showed a reduction in risk of death (HR 0.29; *p* = 0.004), progression to ESRD (HR 0.20; *p* = 0.007), and relapses (HR 0.49; *p* = 0.04) in the CYC and RTX combination group, with rapid GC tapering [20] (doses in Table 1). Similar regimens prospectively studied were shown to be effective and safe [21]. However, as previously mentioned, further trials are needed to confirm the safety and superiority of the combination regimen. Moreover, promising results emerge from studies using low-dose GC. Nevertheless, trials with more statistical power are needed to safely recommend lower dose and shorter GC and CYC regimens [22].

#### 4.1.5. Plasma Exchange

The rationale behind plasma exchange (PLEX) is to reduce circulating serum ANCA levels, as the pathogenic role of ANCA, especially MPO-ANCA, is well supported by animal models [33]. Two major trials studied the use of PLEX in GPA and MPA.

The MEPEX trial compared PLEX to IV pulse methylprednisolone and included patients with GPA or MPA and pauci-immune GN confirmed on renal biopsy with a serum creatinine above 500 *μ*mol/L. At 3 months of follow-up, 69% of participants survived free of dialysis in the PLEX group compared to 49% in the standard pulse therapy (*p* = 0.02). At 12 months, 59% of patients in the plasma exchange group survived free of dialysis versus 43% with pulse therapy (*p* = 0.008), yet no difference was shown in long-term survival at 3 months, 12 months, and 4 years. However, long-term analyses were limited by the low statistical power related to an uneven loss of patients in the study groups [34].

Thirteen years later, the PEXIVAS trial studied four groups of patients in a 2-by-2 factorial design, to assess the efficacy of a reduced-dose GC regimen and PLEX for induction of remission in GPA and MPA. A total of 704 participants with newly diagnosed or severe relapsing GPA or MPA with positive ANCA were included. Severe disease was defined as active GN on biopsy or on urine sediment, with a GFR below 50 mL/min/1.73m^2^, or pulmonary hemorrhage (with compatible pulmonary imaging and either hemoptysis, positive bronchoalveolar lavage, unexplained anemia, or increased diffusing capacity of carbon dioxide). Initial randomization assigned patients to undergo PLEX or not and to receive standard or reduced GC regimen. No survival difference was shown with PLEX; death or ESRD was observed in 28.4% of participants at up to 7 years of follow-up in the PLEX group compared to 31.0% in the control group (*p* = 0.27) [19].

Therefore, urgent PLEX does not need to be routinely performed in most cases of severe AAV. However, caution must be exerted as some subsets of patients with very severe AAV were underrepresented in PEXIVAS, notably patients with diffuse crescentic GN, with creatinine levels above 500 *μ*mol/L, and those requiring hemodialysis and/or affected with severe diffuse pulmonary hemorrhage (DAH). The potential benefit of PLEX in patients with DAH and requiring mechanical ventilation has previously been suggested [35]. Renal response to PLEX might also be predicted by the renal histopathological classification and not solely by the serum creatinine levels [36].

#### 4.1.6. Avacopan

The central role of neutrophil activation in the pathogenesis of AAV is well described [4]. Avacopan, a selective antagonist of the C5a receptor on neutrophils, has anti-inflammatory properties without causing immunosuppression; it blocks the alternative complement cascade in a way that prevents tissue damage while allowing neutrophils to exert their protective functions. Avacopan was compared to prednisone in three trials, two of which are presented here. The small, open-label, and exploratory CLEAR study, a phase 2 trial, enrolled patients with newly diagnosed or severe relapsing GPA or MPA. Participants were randomized in three groups: oral prednisone plus placebo (control group), oral low-dose prednisone plus avacopan, and avacopan without prednisone (doses in Table 2). At 12 weeks of follow-up, treatment with avacopan alone and combined with prednisone was shown noninferior to the control group in achieving disease response (*p* = 0.01 in the avacopan alone group and *p* = 0.002 in the avacopan plus prednisone group) [37].

Prednisone and avacopan were then compared in a randomized, placebo-controlled phase 3 trial, the Avacopan in Antineutrophil Cytoplasmic Antibody- (ANCA-) Associated Vasculitis (ADVOCATE) trial [NCT02994927] [38]. Patients with newly diagnosed or relapsing GPA or MPA were randomized to receive prednisone or the study drug, oral avacopan. Patients from both groups were treated with RTX (375 mg/m^2^ weekly for 4 weeks) or CYC, followed by 1 to 2 mg/kg of AZA. CYC was given orally (2 mg/kg daily for 14 weeks) or IV (15 mg/kg every 2 to 3 weeks for 13 weeks, with a maximum of 1200 mg/dose). Participants in the avacopan group received oral avacopan (30 mg twice daily) for 52 weeks and a placebo prednisone taper. Participants in the prednisone arm received oral doses starting at 60 mg daily, tapered to 0 mg over 20 weeks, and a placebo of avacopan. Remission was defined as BVAS = 0 and discontinuation of GC within 4 weeks before week 26, sustained until week 52. Avacopan was proven noninferior in inducing remission at 26 weeks of follow-up (72.3% in the avacopan group versus 70.1% in the prednisone group; *p* < 0.0001 for noninferiority). Avacopan remained noninferior and even superior to prednisone at 52 weeks for sustained remission (65.7% in the avacopan arm versus 54.9% in the prednisone arm; *p* = 0.0066 for superiority). The study drug was also able to further improve kidney function when compared to prednisone and to reduce cumulative GC toxicity [39].

Considering the importance of GC-induced toxicity in the treatment of AAV, the development of safe reduced GC regimens, as in PEXIVAS, CycLowVas, and ADVOCATE trials, is encouraging and marks the impressive evolution of clinical research in AAV.

### 4.2. Induction of Remission in Nonsevere GPA and MPA

The induction of remission in nonsevere GPA and MPA (without organ- or life-threatening manifestations) usually involves GC in combination with either methotrexate (MTX) or mycophenolate mofetil (MMF). RTX maybe be used; however, it has not been thoroughly investigated in nonsevere GPA and MPA. A lower dose of oral prednisone, 0.5 mg/kg daily (or equivalent), can often be used initially and tapered 2 to 4 weeks after initiation of immunosuppressive therapy. Such a reduced-dose tapering schedule is a reasonable approach for patients with nonsevere AAV and may be adjusted according to clinical response and delay of action of the additional immunosuppressant chosen.

#### 4.2.1. Methotrexate

The Nonrenal Wegener's Granulomatosis Treated Alternatively with Methotrexate (NORAM) trial compared CYC to MTX in newly diagnosed early systemic ANCA-associated vasculitis without critical organ manifestations. Both treatments were administered for 12 months. The remission rate at 6 months of this unblinded trial was noninferior with MTX compared to CYC (89.8% versus 93.5%, *p* = 0.041). However, in patients with extensive disease or pulmonary involvement, time to achieve remission was longer with MTX as opposed to CYC. At 18 months, relapses were more common in patients treated with MTX than CYC (69.5% versus 46.5%; *p* = 0.023), which demonstrated at that time the importance of immunosuppressive therapy beyond 12 months [40]. MTX may therefore be used for induction of remission of nonsevere AAV, at weekly doses of 0.3 mg/kg orally or subcutaneously, with a maximum dose of 25 mg. Maintenance of remission therapy is recommended in most cases once remission is achieved and will be further discussed below [12, 14–16].

#### 4.2.2. Mycophenolate Mofetil

MMF, an immunosuppressant that acts selectively on lymphocytes, is used for various autoimmune diseases and for organ transplantation [41]. In the Mycophenolate Mofetil versus Cyclophosphamide for Remission Induction in AAV (MYCYC) trial, MMF was compared to CYC in newly diagnosed GPA or MPA. Participants with life-threatening vasculitis, rapidly progressive GN, or a GFR below 15 mL/min/m^2^ were excluded. At induction of remission, participants received standard GC therapy and either 2000 mg of MMF daily, which was increased to 3000 mg if remission was not achieved at 4 weeks, or 15 mg/kg of IV CYC pulse every 2-3 weeks. Once remission was achieved, both groups received AZA 2 mg/kg daily as maintenance therapy. MMF was noninferior to CYC to induce remission at 6 months (67% vs. 61%, respectively, risk difference of 5.7%, 90% CI (-7.5–19%)). However, patients receiving MMF as an induction therapy had a higher relapse rate in the following 6 months after achieving remission (*p* = 0.049) [42]. Hence, MMF, up to 3000 mg daily, can be considered in induction of remission of patients with nonsevere AAV.

## 5. Maintenance of Remission Therapy

**Figure 3:**
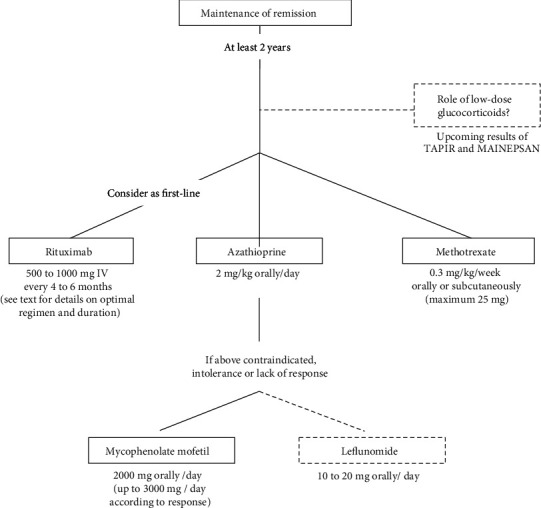
Suggested algorithm of maintenance therapy in severe GPA and MPA.

Once remission is achieved, maintenance of remission (Figure 3) is important to prevent disease relapses. Vasculitis disease flares after achieving remission may occur in 5-50% of patients on maintenance therapy and in up to 80-90% of patients if no maintenance therapy is initiated [13]. Many factors, several still unknown, influence the risk of disease flare; patients with GPA, previous history of relapse(s), and PR3-ANCA positive are more likely to relapse (again) than patients with MPA [7] or with MPO-ANCA [7]. The most widely used therapies for maintenance of remission include MTX, AZA, and RTX. In certain cases, MMF or leflunomide (LEF) may be used. The role of prednisone in the maintenance of remission will also be discussed.

### 5.1. Methotrexate, Azathioprine, Mycophenolate Mofetil, and Leflunomide

The safety of MTX in maintenance therapy was evaluated in the Wegener's Granulomatosis–Entretien (WEGENT) trial. Patients with newly diagnosed GPA or MPA and positive serologic testing or biopsy were enrolled in this study that compared MTX to AZA (doses in Table 2) for maintenance of remission. At median follow-up of 29 months, no difference in adverse reactions or relapses was noted between MTX and AZA, with 33% and 36% of participants relapsing, respectively (*p* = 0.71) [43]. Longer follow-up of the same cohort of patients evaluated the rate of relapses or adverse events after discontinuation of the two drugs [44]. No significant difference was proven in relapse-free survival (33.5% in the MTX group versus 26.3% in the AZA group; *p* = 0.29) and in overall survival (79.9% in the MTX group versus 75.1% in the AZA group; *p* = 0.56) at 10 years of follow-up after maintenance treatment.

The Cyclophosphamide versus Azathioprine as Remission Maintenance Therapy for ANCA-Associated Vasculitis Study (CYCAZAREM) compared continuous CYC versus a switch to AZA for maintenance of remission (doses in Table 2). Participants with newly diagnosed generalized AAV initially all received prednisone and oral CYC (2 mg/kg per day). Once remission was achieved between 3 to 6 months after treatment initiation, patients were randomized to continue oral CYC (1.5 mg/kg per day) or replace it with AZA (2 mg/kg daily) for 12 months. All participants were switched to AZA after 12 months. No difference in relapse rate was noted at 18 months' follow-up (15.5% for AZA and 13.7% for CYC; *p* = 0.65) [45]. Because of the potential adverse effects of CYC, switching to AZA for maintenance therapy after induction of remission achieved with CYC was deemed safer and appropriate [12, 14–16].

The duration of maintenance therapy with AZA was studied in the prolonged Remission-Maintenance Therapy in Systemic Vasculitis (REMAIN) trial. A significant reduction of relapse rates was noted with 48 months of treatment compared to 24 months only, of 22% and 63%, respectively (*p* < 0.0001). Interestingly, ANCA positivity at randomization was a predictor of relapse [46].

Subsequently, AZA was compared to MMF for maintenance therapy in the International Mycophenolate Mofetil Protocol to Reduce Outbreaks of Vasculitides (IMPROVE) trial. All patients achieved remission with GC and CYC and then were randomized to receive daily AZA (2 mg/kg for 12 months, 1.5 mg/kg for 6 months, and 1 mg/kg until month 42) or daily MMF (2000 mg for 12 months, 1500 mg for 6 months, and 1000 mg until month 42). At median follow-up of 39 months, an increased incidence of first relapses occurred in the MMF group compared to AZA (HR 1.69; 95% CI 1.06-2.07; *p* = 0.03). Moreover, a higher incidence of first major relapse was shown in the first group compared to the latter (HR 2.14; 95% CI 0.99-4.64; *p* = 0.054) [47]. Consequently, AZA is considered a better option than MMF to maintain remission in patients with GPA or MPA.

LEF exerts anti-inflammatory and immunomodulatory effects through activation of p53, resulting in the inhibition of the proliferation of lymphocytes [48]. LEF was compared to MTX for maintenance of remission. Patients with generalized GPA were enrolled in a RCT to receive MTX or LEF after induction therapy with CYC. Participants in the LEF arm had significantly fewer major relapses (*p* = 0.037) in the 2 years following remission [49]. More recently, LEF was described in a retrospective study as an induction and maintenance therapy in 93 participants with different vasculitides, including 45 patients with GPA and 8 patients with MPA. Most patients had low-dose prednisone and 89% had an active disease at treatment initiation. In patients with GPA, 69% achieved remission at 6 months [50]. Hence, although evidence is limited, LEF may be considered as an alternative agent for maintenance therapy in AAV [51].

### 5.2. Rituximab for Maintenance of Remission

Evidence around RTX in maintenance therapy comes mainly from a series of trials called the MAINRITSAN trials (Table 2). The first Maintenance of Remission using Rituximab in Systemic ANCA-Associated Vasculitis (MAINRITSAN) study included newly diagnosed, severe relapsing GPA or MPA, or renal-limited ANCA-associated vasculitis. Patients received the standard induction treatment with prednisone and CYC until remission and then were randomized to receive either RTX for 18 months or AZA for 22 months. RTX dosing was 500 mg IV at days 0 and 14 and then at months 6, 12, and 18. Oral AZA was given at 2 mg/kg daily for 12 months, 1.5 mg/kg daily for 6 months, and then 1 mg/kg daily for 4 months, for a total of 22 months. At 28 months of follow-up, major relapses occurred less frequently in the RTX group compared to the AZA group (5% versus 29%, respectively; *p* = 0.002) [52]. The same patients were further followed for 60 months; improved survival was noted in the RTX group compared to AZA, 100% versus 93%, respectively (*p* = 0.045). Moreover, an increased major relapse-free survival was shown in the RTX group (49.4% versus 71.9%, respectively; *p* = 0.003) [53].

Two different regimens of RTX for maintenance therapy were compared in the MAINRITSAN-2 trial, using a fixed or individualized schedule. Participants in the fixed-dose group received RTX every 6 months, like the first MAINRITSAN study. In the individualized schedule group, participants were treated according to CD19+ B lymphocytes and ANCA titers. They received the first infusion of RTX; additional infusions were given only if CD19+ B cell counts were superior to 0/mm^3^, if ANCA titers increased (2-fold rise) or became positive (when previously negative), when measured every three months. A median of five and three infusions in two years were received in the fixed and the individualized schedule groups, respectively. At 28 months of follow-up, no significant difference in relapse rate was shown between treatment regimens (9.9% for fixed schedule versus 17.3% for individualized; *p* = 0.22) [54].

Patients from the MAINRITSAN-2 trial were subsequently enrolled in the MAINRITSAN-3 trial, if they were in remission at the end of the former study follow-up period. Long-term use of RTX was studied by giving additional 500 mg IV RTX doses every 6 months for 2 years more versus placebo infusions; 2 versus 4 years of maintenance treatment with RTX were therefore compared. An increased relapse-free survival was associated with prolonged RTX treatment and no major relapses or rise in adverse effects was seen in this group. At 56 months, relapse-free survival rates in RTX versus placebo groups were 96% and 74%, respectively (*p* = 0.008), with no difference in severe adverse effects [55].

RTX was also studied specifically for maintenance of remission in patients with GPA or MPA following a relapse. The RITAZAREM trial [NCT01697267] compared RTX (1000 mg IV every 4 months) versus AZA for maintenance therapy. At 24 months, 13% of patients receiving RTX relapsed versus 38% for patients treated with AZA (HR 0.35, *p* < 0.001) [56].

In summary, at this time, RTX should be the preferred agent to maintain remission in patients with AAV, as it was shown superior to AZA in newly diagnosed patients and those with relapsing disease [5]. In patients at higher risk of relapse, RTX should probably be continued for 4 years. Some vasculitis experts prefer initiating RTX using the fixed schedule regimen for the first 2 years and then transitioning to the individualized RTX regimen (based on ANCA and CD19+ B lymphocyte done every 3 months). More studies are needed to better individualize the treatment regimen, based on each patient and disease characteristics.

### 5.3. Role of Low-Dose Glucocorticoids to Prevent Relapses

The role of prolonged low-dose oral GC (prednisone 5 mg daily for example) has largely been questioned regarding its safety and efficacy for the maintenance of remission. The Assessment of Prednisone in Remission Trial (TAPIR) [NCT01933724] is aimed at determining the rate of relapse at 6 months in patients continuing low-dose prednisone for maintenance therapy compared to its discontinuation. More precisely, participants enrolled in this study are in remission after a flare, which happened within the previous 12 months, and on daily prednisone doses of 5 mg at randomization. Patients are then randomized to continue the same dose or to taper it down to 0 mg within a month. The rate of relapse is then assessed at 6 months after randomization [57]. Results of this trial, still recruiting, will help to give us insight on the use of GC to maintain remission. In France, the MAINEPSAN trial enrolls GPA or MPA patients in remission with maintenance RTX regimen. The diagnosis must have been made more than 12 months prior to inclusion and patients must have received GC for 12 months before enrollment. All participants are treated with 5 to 10 mg of daily prednisone within 35 days prior to randomization. Patients are then randomized to continue 5 mg of daily prednisone until week 52 or to discontinue prednisone within a month, using a weekly taper of 1 mg. Relapse-free survival will be assessed at week 120 of randomization.

### 5.4. Other Therapies: Belimumab, Abatacept, and Trimethoprim-Sulfamethoxazole

More recently, belimumab was studied as a maintenance therapy in GPA and MPA. Belimumab is a human monoclonal IgG*λ* antibody against B lymphocyte stimulator (BLyS). In the Belimumab in Remission of Vasculitis (BREVAS) trial, this drug was compared to placebo for maintenance of remission in patients with newly diagnosed or relapsing severe GPA or MPA. All patients received a standard remission induction regimen, which included high-dose GC plus CYC or RTX. After remission was achieved, participants were randomized to receive either AZA combined with belimumab or AZA with placebo. At 12 months of follow-up, no difference was seen in relapse rate between the belimumab and placebo treatment arms, with a HR of 0.88 (*p* = 0.821). Interestingly, in the subgroup of patients who received RTX as an induction therapy, none of the patients relapsed when belimumab was administered in the maintenance therapy regimen, as opposed to patients who only received AZA [58]. As RTX is known to increase circulating BLyS levels, blockage of this stimulator by belimumab may improve disease control and prevent relapses [59]. The Rituximab and Belimumab Combination Therapy in PR3 Vasculitis (COMBIVAS) trial [NCT03967925] is currently ongoing in the United Kingdom and compares treatment regimens with RTX alone or combined with belimumab in patients with PR3-ANCA-positive AAV. Results of this trial are expected in 2023 [60].

Another drug studied in AAV treatment is abatacept. This molecule binds the CTLA4 domain linked to IgG1 and blocks the action of CD28, therefore preventing antigen-presenting cells to activate T cells [61]. In relapsing, nonsevere GPA, the Abatacept for the Treatment of Relapsing, Nonsevere, Granulomatosis with Polyangiitis (ABROGATE) trial [NCT02108860] is currently studying the efficacy of the drug to achieve GC-free remission. Results of this trial are expected in 2023.

Treatment with trimethoprim-sulfamethoxazole (TMP-SMX) can be considered in a subset of patients with GPA with upper airway involvement. The use of TMP-SMX for maintenance therapy is possible, most often as an adjuvant treatment or following the many years of immunosuppression, but is limited due to the higher doses required (twice a day), frequent common side effects (nausea, fatigue, and rash), and potential increased risk of toxicity when used with methotrexate [62].

## 6. Conclusion

The treatment of GPA and MPA is rapidly evolving. As mortality and remission rates have significantly improved over the years, our attention is now focused on how to achieve rapid and sustained remission while minimizing treatment toxicity. As these diseases are heterogeneous in their presentation, further research on individualization of treatment according to disease subtype, genetic factors, and risk of relapse will be necessary to truly offer a state-of-the-art therapy.

## 7. Take-Home Messages

### 7.1. Induction of Remission

- First-line agents for induction of remission in severe GPA and MPA include cyclophosphamide or rituximab, along with glucocorticoids
- Rapid reduction of glucocorticoids is safe and should be considered for induction of remission in severe GPA and MPA
- Avacopan, if confirmed safe and cost-effective, may become an alternative to GC and lead to a GC-free regimen of AAV
- Plasma exchange is no longer routinely recommended in most patients with severe GPA and MPA; it has not been proven effective in terms of survival and reduction of ESRD risk in patients with severe disease. PLEX can still be considered in carefully selected patients in conjunction with vasculitis expert opinions

### 7.2. Maintenance of Remission

- Rituximab should be considered as the first-choice agent for maintenance of remission; azathioprine and methotrexate are appropriate alternatives if rituximab is unavailable or contraindicated
- Longer treatment duration with rituximab for maintenance of remission reduces relapses in severe GPA and MPA, but it is unclear to date whether all patients, or only some subsets, would benefit of 4-year duration of repeated, systematic RTX infusions
- Leflunomide and mycophenolate mofetil can be used when RTX, MTX, and AZA are contraindicated or not tolerated
- The role of low-dose prednisone in maintenance of remission is not definitely demonstrated or established (ongoing studies)

## Figures and Tables

**Table 1:** Induction therapy studies of ANCA-associated vasculitis discussed in this article.

	Trial	Treatment	Study type	Population	Treatment	Outcomes
CYC	CYCLOPS trial (2009) [25]	CYC daily oral vs. CYC IV	RCT	Newly diagnosed severe GPA or MPA with renal involvement	IV CYC: 15 mg/kg weeks 0, 2, and 4 then q3 weeks continued for 3 months after remission (maximum 1200 mg IV/dose) Oral CYC: 2 mg/kg/day until remission then 1.5 mg/kg/day x 3 months (maximum oral dose of 200 mg)	At 9 months: no difference in time to remission with a lower rate of leukopenia and reduced cumulative dose with IV CYC (*n* = 149)
	CYCLOPS trial—long term (2012) [26]					At 4.3 years of median follow-up: significantly lower relapses with oral CYC; no difference in mortality or renal survival (*n* = 134)
MTX	NORAM study (2005) [40]	MTX vs. CYC	RCT	Newly diagnosed GPA or MPA without critical organ manifestations (*n* = 100)	Oral CYC: 2 mg/kg/day until remission (3-6 months) and then 1.5 mg/kg/day until month 10 and discontinued by month 12 Oral MTX: 20-25 mg/week for 12 months and then discontinued	At 6 months: MTX noninferior for remission rate; delayed remission in patients with more extensive disease At 18 months: increased relapses with MTX
MMF	MYCYC (2019) [42]	CYC vs. MMF	RCT	Newly diagnosed GPA or MPA excluding severe disease (*n* = 140)	CYC: IV pulse CYC 15 mg/kg q2-3 weeks for 6 months MMF: 2 g/day increased to 3 g/day (if required) for 3-6 months Both groups received AZA 2 mg/kg daily as maintenance therapy	At 6 months: MMF noninferior for remission At 18 months: more disease relapses with MMF compared to CYC, especially in PR3-ANCA patients
Rituximab	RITUXVAS trial (2010) [28]	RTX vs. CYC	RCT	Newly diagnosed generalized GPA or MPA with renal involvement (*n* = 44)	RTX: 375 mg/m^2^ IV weekly x 4 (patients received IV CYC 15 mg/kg x 2 doses at weeks 0 and 3) CYC: 15 mg/kg IV × 3 to 6 months and then AZA	At 12 months: no significant difference in sustained remissions, SAE, and deaths
	RAVE trial (2010) [29]	RTX vs. CYC	RCT	Newly diagnosed or severe relapse of GPA or MPA with positive ANCA (*n* = 197)	RTX: 375 mg/m^2^ IV q week x 4 Oral CYC: 2 mg/kg/day x 3-6 months until remission and then AZA (2 mg/kg/day)	At 6 months: RTX noninferior for remission (BVAS = 0 and successful completion of prednisone tapering) At 6 months: RTX superior for relapsing vasculitis or PR3-positive patients
	RAVE trial—long term (2013) [30]					At 18 months: RTX noninferior for remission (BVAS = 0)
Reduced GC	Glucocorticoid (Pepper et al.) (2018) [22]	Standard vs. reduced GC	Multicenter cohort study	Active MPO- or PR3-ANCA vasculitis or ANCA-negative pauci-immune GN (*n* = 49)	Induction therapy with two doses of RTX and three months of low-dose pulse IV CYC followed by short course (1 to 2 weeks) of GC	Similar outcomes to a matched population from the EUVAS trials, with lower exposure to CYC and GC
	CycLowVas (2019) [20]	CYC+RTX+reduced GC	Single-center cohort study	Renal ANCA-associated vasculitis (excluding alveolar hemorrhage, cerebral vasculitis, creatinine > 500 *μ*mol/L) (*n* = 66)	RTX: 1000 mg IV at day 0 and day 14 CYC: 10 mg/kg IV weekly for 2 weeks, followed by 500 mg IV weekly for 4 weeks (total of 6 doses) Reduced prednisone: rapid taper reaching 12.5 mg/day at week 12	At median follow-up of 56 months: reduced risk of death, progression to ESRD, and reduced relapses
Plasma exchange	MEPEX trial (2007) [34, 63]	PLEX vs. MP	RCT	Newly diagnosed severe renal GPA or MPA (*n* = 137)	PLEX: 7 treatments (60 mL/kg) in 14 days followed by standard therapy (CYC+prednisone) MP: 1000 mg IV per day x 3 days followed by standard therapy	At 3 and 12 months: more survivors free of dialysis with plasma exchange No difference in long-term survival at 3 months, 12 months, and 4 years
	PEXIVAS trial (2020) [19]	PLEX and reduced GC	RCT	Newly diagnosed or severe relapse of GPA or MPA with positive ANCA (*n* = 704)	Four study groups in a 2-by-2 factorial design receiving (i)-PLEX (7 treatments in 14 days) vs. no PLEX (ii)-Standard GC doses (PEXIVAS) vs. reduced GC (PEXIVAS-reduced)	Up to 7 years of follow-up, no difference in death from any cause or ESRD
Avacopan	CLEAR trial (2017) [37]	Avacopan vs. prednisone	RCT	Newly diagnosed or relapsing GPA or MPA (*n* = 67)	All groups: standard induction therapy (CYC or RTX) Control group: avacopan placebo+prednisone 60 mg daily Study group: avacopan 30 mg twice daily+prednisone 20 mg daily Study group: avacopan 30 mg twice daily, no prednisone	At 12 weeks: avacopan treatment with or without prednisone was noninferior to the control group in achieving disease remission
	ADVOCATE trial *Abstract* (2020) [39]	Avacopan vs. prednisone	RCT	ANCA MPA or GPA patients receiving RTX or CYC/AZA (*n* = 330)	All patients: RTX (375 mg/m^2^ weekly for 4 weeks) or CYC orally (2 mg/kg daily for 14 weeks) or IV (15 mg/kg every 2 to 3 weeks for 13 weeks, maximum of 1.2 g/dose), followed by 1 to 2 mg/kg of AZA at week 15 Prednisone: 60 mg (taper over 21 weeks) Avacopan: 30 mg orally twice daily for 52 weeks	At week 26: noninferior remission rate in the avacopan group compared to prednisone At 52 weeks: noninferiority and superiority for sustained remission in the avacopan arm
Abatacept	*ABROGATE*	Abatacept vs. placebo	RCT	Relapsing nonsevere GPA (*n* = 66)	Abatacept: 125 mg sc q week vs. abatacept placebo	*Results expected in 2023*

Abbreviations: ad = until; ANCA = antineutrophil cytoplasmic antibody; AZA = azathioprine; CYC = cyclophosphamide; ESRD = end-stage renal disease; GC = glucocorticoid; GFR = glomerular filtration rate; GN = glomerulonephritis; GPA = granulomatosis with polyangiitis; IV = intravenous; MMF = mycophenolate mofetil; MP = methylprednisolone; MPA = microscopic polyangiitis; MTX = methotrexate; q = every; PLEX = plasma exchange; RCT = randomized controlled trial; RTX = rituximab; SAE = severe adverse events; sc = subcutaneous; vs. = versus; x = times.

**Table 2:** Maintenance therapy trials of ANCA-associated vasculitis discussed in this article.

	Trial	Therapy	Study type	Population	Doses	Outcomes
CYC	CYCAZAREM trial (2003) [45]	CYC vs. AZA	RCT	Newly diagnosed generalized ANCA-associated vasculitis after induction with GC and oral CYC. (*n* = 144)	CYC: 1.5 mg/kg/day for 12 months AZA: 2 mg/kg/day for 12 months Both arms received after AZA until month 12	No difference in relapse and adverse events at 18 months of follow-up
MMF	IMPROVE trial (2010) [47]	AZA vs. MMF	RCT	Newly diagnosed GPA or MPA after induction with GC and CYC. (*n* = 156)	AZA: 2 mg/kg/day for 12 months, then 1.5 mg/kg/day for 6 months, and 1 mg/kg/day until month 42 MMF: 2000 mg/day for 12 months, then 1500 mg for 6 months, and 1000 mg until month 42	At median follow-up of 39 months: increased incidence of first relapse in the MMF group compared to AZA; increased incidence of first major relapse in the MMF group compared to AZA
MTX	WEGENT trial (2008) [43]	MTX vs. AZA	RCT	Newly diagnosed GPA or MPA with positive serologic or histological ANCA, after induction with GC and CYC	AZA: 2 mg/kg/day for 12 months MTX: 0.3 mg/kg/week (oral or subcutaneous), progressively increasing to 25 mg/week for 12 months	At median follow-up of 29 months: No difference in adverse reactions and relapses (*n* = 126)
	WEGENT trial—long term (2016) [44]					At 10 years: no significant difference in relapse-free survival (*n* = 112)
AZA	REMAIN trial (2017) [46]	Prolonged AZA treatment for maintenance	RCT	Newly diagnosed GPA or MPA or renal-limited vasculitis after induction with GC and CYC (*n* = 117)	Maintenance with AZA and prednisone low dose for 24 vs. 48 months	Significant reduction of relapse with 48 months of treatment compared to 24 months; ANCA positivity at randomization associated with relapse risk
Rituximab	MAINRITSAN trial (2014) [52]	RTX vs. AZA	RCT	Newly diagnosed or relapse of severe GPA or MPA or renal-limited vasculitis in complete remission after induction therapy with GC and CYC (*n* = 115)	RTX: 500 mg IV at days 0 and 14 and then at months 6, 12, and 18 (total 18 months) AZA: 2 mg/kg/day for 12 months, 1.5 mg/kg/day for 6 months, and then 1 mg/kg/day for 4 months (total: 22 months)	At 28 months of follow-up: less relapses with RTX
	MAINRITSAN-1—60 months (2018) [53]					At 60 months: improved survival and increased major relapse-free survival with RTX
	MAINRITSAN-2 (2018) [54]	Fixed RTX vs. individualized	RCT	Newly diagnosed or relapsing severe GPA or MPA in complete remission after induction therapy with GC and CYC or RTX (*n* = 162)	Fixed: 500 mg IV at days 0 and 14 and then at 6, 12, and 18 months Individualized: 500 mg IV at randomization and then reinfusion only if reappearance of CD19 or ANCA or increased titer of ANCA; measured every 3 months, until month 18	Median of 5 vs. 3 infusions in 2 years, respectively At 28 months of follow-up: no significant difference in relapse rate; ANCA and CD19 measured every 3 months do not predict relapse
	MAINRITSAN-3 (2020) [55]	RTX 2 vs. 4 years	RCT	Newly diagnosed or relapsing severe GPA or MPA in complete remission following the completion of MAINRITSAN-2 trial (*n* = 97)	Four additional 500 mg IV doses of RTX: at inclusion, months 34, 40, and 46 vs. placebo	At 56 months: relapse-free survival rates superior with RTX with no difference in severe adverse events
	RITAZAREM *Abstract* (2019) [56]	RTX vs. AZA	RCT	Maintenance therapy after a major relapse of GPA or MPA after induction with GC+RTX (*n* = 170)	RTX: 1000 mg IV every 4 months x 5 doses AZA: 2 mg/kg/day	At 24 months of follow-up: RTX superior to AZA to prevent relapses
Belimumab	BREVAS trial (2019) [58]	Belimumab vs. placebo	RCT	Newly diagnosed or relapsing severe GPA or MPA after induction with GC and either CYC or RTX (*n* = 105)	All patients: AZA (2 mg/kg/day) and low-dose GC Belimumab: 10 mg/kg IV on days 0, 14, and 28 and then every 28 days vs. placebo of belimumab	At 12 months: no difference in vasculitis relapse No relapsing disease in patients receiving RTX followed by belimumab
	*COMBIVAS*	Belimumab+RTX vs. RTX alone	RCT	Patients with PR3-positive AAV (*n* = 30)	Rituximab: 1 g IV x 2 doses (all patients) Belimumab group: 200 mg sc q week	*Results expected in 2023*
Prednisone	*TAPIR trial*	Low-dose prednisone	Open label	GPA in remission (*n* = 60)	All patients tapered to 5 mg of daily prednisone and then randomized Prednisone: 5 mg daily No prednisone: taper to 0 mg	*Results pending* Endpoint: rate of relapse being the endpoint at 6 months after randomization
	*MAINEPSAN trial*	Low-dose prednisone	RCT	Patients with GPA or MPA in remission, 12 months following induction therapy	Prednisone: continue 5 mg daily for 12 months No prednisone: taper to 0 mg in 1 month	*Recruiting in France*

Abbreviations: ANCA = antineutrophil cytoplasmic antibody; AZA = azathioprine; CYC = cyclophosphamide; GC = glucocorticoids; GPA = granulomatosis with polyangiitis; GFR = glomerular filtration rate; GN = glomerulonephritis; IV = intravenous; MMF = mycophenolate mofetil; MPA = microscopic polyangiitis; q = every; RCT = randomized controlled trial; RTX = rituximab; sc = subcutaneous.

## Data Availability

All data presented in this review article has been published as original articles or abstracts and available online.
